# Changes in dietary and lifestyle behaviors and mental stress among medical students upon Ramadan diurnal intermittent fasting: a prospective cohort study from Taif/Saudi Arabia

**DOI:** 10.1186/s12889-023-16385-1

**Published:** 2023-07-31

**Authors:** Mohammed I. Alotaibi, Ghadir Elsamad, Abdulaziz N. Aljardahi, Ahmed N. Alghamdi, Abdulrahman I. Alotaibi, Hibah M. Alorabi, Khalid M. Alzahrani, Ahmed S. Abdel-Moneim

**Affiliations:** 1 College of Medicine, Taif University, Al-Taif 21944, Taif, Saudi Arabia; 2 Translational Health Sciences, University of Bristol, Bristol, BS1 3NY UK; 3 King Abdulaziz Specialist Hospital, Al-Taif 21944, Taif, Saudi Arabia

**Keywords:** Ramadan, Intermittent fasting, Lifestyle, Stress, Academic performance

## Abstract

**Background:**

Intermittent fasting (IF) is a popular dietary plan for weight loss. In fact, fasting is a common practice in different religions such as Buddhism, Hinduism, Judaism, Christianity, and Islam. During the month of Ramadan, more than 1.5 billion Muslims worldwide fast from dawn to sunset. Ramadan diurnal intermittent fasting (RDIF) has health benefits, including a reduction in cardiovascular disease (CVD) risk and an improvement in mood. However, little is known about the effects of RDIF on lifestyle behaviors, such as regular exercise, consuming healthy diet, and avoiding harmful substances, as well as mental stress, and academic performance in high school and university students.

**Methods:**

In this prospective cohort study, two self-reported questionnaires were sent one week before and during the last week of Ramadan (April 2022; Ramadan 1443 in Hijri Islamic Calendar) to assess changes in lifestyle, perceived stress, and academic achievement of medical students at Taif University in Taif city, Saudi Arabia. Healthy lifestyle components data were collected to calculate healthy lifestyle scores, including body mass index, physical activity, adherence to a Mediterranean diet, smoking status, and sleep duration.

**Results:**

RDIF was associated with a healthier lifestyle in both female and male participants (pre-RDIF mean score: 2.42 vs post-RDIF mean score: 2.74; statistical power = 0.99; *P*-value < 0.05). They were more active and adherent to the Mediterranean diet during RDIF. Additionally, the post-RDIF smoking rate declined by 53.4%. Male participants showed higher perceived stress scores during RDIF (pre-RDIF mean score: 19.52 vs post-RDIF mean score: 22.05; *P*-value < 0.01). No changes in academic performance were observed upon RDIF.

**Conclusion:**

Medical students show healthier dietary and lifestyle behaviors and their academic performance is not affected during RDIF. However, perceived stress is higher among male students.

**Supplementary Information:**

The online version contains supplementary material available at 10.1186/s12889-023-16385-1.

## Background

Ramadan is the holy month in Islam during which Muslims fast daily. It is a form of intermittent fasting (IF) in which Muslims must abstain from food and all fluid intake from dawn to sunset. Ramadan diurnal intermittent fasting (RDIF) is safe for all healthy individuals and can be associated with a considerable reduction in body weight and improvement in cardiometabolic health [1, 2]. In contrast to other forms of IF, RDIF is a religious fasting, associated with spiritual and social aspects, which could have beneficial effects on mental health as well. RDIF is associated with lower stress, anxiety, and depressive symptoms [3]. Nevertheless, changes in meal timing and lifestyle habits during RDIF may affect circadian rhythm and lead to changes in sleep, stress levels, and cardiometabolic health [4–6]. Furthermore, disrupted cortisol levels have been reported during Ramadan among healthy young adults, which might be attributed to changes in sleep and feeding behavior in modern fasting practices [7]. However, a recent study showed no correlation between RDIF and disordered eating habits in adolescents [8]. Additionally, sleep patterns and sleep disorders were assessed objectively using polysomnography in healthy adult male Muslims before and after Ramadan fasting. RDIF showed no effects on sleep stages or daytime sleepiness although the rapid eye movement (REM) sleep phase was reduced with RDIF [9]. However, there is a paucity of research investigating the impact of RDIF on lifestyle habits, such as diet and sleep, as well as stress levels among university students.

Lifestyle medicine is a growing field of medicine that focuses on the prevention and treatment of diseases caused by unhealthy lifestyle factors. Undoubtedly, daily habits and practices have a significant impact on short-term and long-term health conditions and quality of life [10]. The key elements of a healthy lifestyle include consuming a nutritious diet, being physically active, maintaining a healthy weight, sleeping well, as well as avoiding smoking and alcohol intake. Modification of these factors plays a fundamental role in promoting physical and mental well-being as well as preventing chronic illnesses [11, 12]. Healthy lifestyles such as consuming a diet rich in fruits and vegetables, maintaining an ideal body mass index (BMI), and exercising regularly are associated with reduced incidence of dementia and chronic diseases, including diabetes and vascular diseases [13]. In young adulthood, a healthy lifestyle is associated with a lower risk of developing cardiovascular disease by middle age [14]. In addition, a growing body of evidence suggests an association between a healthy lifestyle and academic performance, especially in children and adolescents [15–18]. Moreover, academic impairment is associated with an unhealthy lifestyle in university students [19]. However, those who fast during Ramadan tend to be less alert during the day, whereas more active at night [3]. RDIF has shown a negative impact on the academic performance of Muslim students in a non-Muslim country while a recent study reported that increases in student performance are associated with longer fasting hours in Muslim countries [20, 21]. However, there’s no evidence of impaired academic achievement in Muslim countries, where study schedules might be adjusted to reduce the burden on students during fasting.

Mental stress is common among medical students and may adversely affect their academic performance as well as eating and sleep [22–24]. For example, the prevalence of stress among medical students in Saudi Arabia ranges between 50–60% [25–27]. Although they are expected to adopt a healthy lifestyle, unhealthy eating habits, such as consuming fast food and not exercising regularly, are common among medical students [28, 29]. Reductions in physical activity, diet quality, and general health were reported among medical students [30, 31]. However, there is a lack of studies assessing stress and lifestyle changes among medical students and their impact on academic achievement during Ramadan. In our study, we hypothesized that RDIF is associated with a healthier lifestyle and changes in mental stress in medical students. Thus, we aimed to evaluate the impact of RDIF on dietary and lifestyle habits, mental stress, and academic performance among medical students, using self-reported questionnaires. The findings of this study encourage further research for promoting well-being and improving the academic performance of the students who fast during Ramadan or follow intermittent fasting schedules for health benefits.

## Methods

### Aim and study design

This is a prospective study to evaluate the impact of RDIF on dietary and lifestyle habits, stress levels, and academic performance among medical students at Taif University in Saudi Arabia (April 2022; Ramadan 1443 in Hijri Islamic Calendar). Two questionnaires were sent to them at two different times: just a few days before Ramadan and during the last week of Ramadan. The average fasting duration is approximately 14 h and 28 min with an average temperature ranging from 13–35 °C and a relative humidity of 20–40%. Since this is a prospective study, participants were asked to provide their university ID numbers to make sure that the same students had participated throughout the study. Both male and female students from 1^st^ to 6^th^ year had been invited. The study included healthy students and excluded pregnant, lactating mothers, and those with a history of any psychological problems or admitted to a hospital for any reason during Shaban (the last month before Ramadan) and/or Ramadan.

### Questionnaire

Two questionnaires were sent to at least 300 medical students at Taif University at two different times. Surveys were created using Google Forms including self-reported questionnaires. Since English is the primary language of education in the medical school at Taif University, questionnaires were developed in English. They included questions about demographic data, self-reported or perceived body weight (kg) and height (cm), dietary habits, physical activity, smoking, and sleep. Students were instructed on how to measure their weight and height accurately. Body mass index (BMI) was calculated. Healthy lifestyle score was calculated using data obtained for BMI, adherence to a Mediterranean diet, physical activity, smoking status, and sleep duration. The total healthy lifestyle score ranges from 0 to 5, with higher scores indicating healthier dietary and lifestyle behaviors (BMI < 25 = 1, adherence to MMDS = 1, moderate/vigorous physical activity ≥ 3 times(≥ 150 min)/week = 1, non-smoking = 1, and at least 7–8 h total sleep hours per day = 1). Modified Mediterranean diet scores (MMDSs) were calculated using a self-reported questionnaire containing 14 questions related to a Mediterranean diet (Supplementary Table 1). This has been validated and used before [32]. Stress was assessed using the Perceived Stress Scale (PSS), a widely used stress assessment tool that includes ten questions (Supplementary Table 2) [33]. The PSS-10 questionnaire has been validated to evaluate the perceived stress among university students [34]. The PSS score ranges from 0–40, where a score of 0–13 indicates low stress, a score of 14–26 is moderate, and a score of 27–40 is high perceived stress. PSS scores were obtained for the month before RDIF and at the end of Ramadan.

### Statistical analyses

Since this is the first study to assess lifestyle scores during Ramadan, sample size was roughly estimated based on previous studies that showed associations between RDIF and dietary changes [35], cardiometabolic health [36] or mental well-being [37]. We also considered studies that assessed lifestyle scores and risk of cancer [38] and cardiovascular disease [39]. We concluded that at least 63 samples were required with α = 0.05 and power = 0.08. To increase the power, we invited around 302 students (female = 150, male = 152).

Means and standard deviations (SD) of dietary and lifestyle scores and PSS scores were calculated. Changes in scores were measured to evaluate the differences between the two time points. Comparisons were also performed based on sex and academic year. Additionally, changes in dietary lifestyle score components during RDIF were assessed. Academic performance was also evaluated before and at the end of RDIF by collecting the last exam grades. Taif University grading system was used, and grades were categorized into: A + /A (excellent), B + /B (very good), C + /C (good), D + /D (acceptable), and F (failed). Differences in academic performance before and at the end of RDIF were also calculated. Statistical analysis was performed using Prism 8 (GraphPad Software), and Stata software was used for sample size calculation and statistical power analysis. The significant difference was tested by paired t-test for overall sample, Student's t-test for comparing sex groups, McNemar or Chi-square test for comparing categorical data or ANOVA and a Tukey test or Sidak posthoc test for comparing 3 or more groups. Regression analysis was performed to estimate the effect of the academic year on dietary and lifestyle scores (the outcome variable). Additionally, linear regression was done to predict the impact of dietary and lifestyle components on perceived stress scores.

## Results

### Sample characteristics

A total number of 220 (109 female and 111 male medical students) participated in this study. Most of the participants were in their first, second, or third year (27.7%, 25.9%, and 25%, respectively). In contrast, 4^th^, 5^th^, and 6^th^-year medical students were less initiative and some of them filled out only the first survey. Those who didn’t complete all the surveys were excluded (30 students). Most of our respondents were healthy (90.9% with no chronic illnesses), non-smokers (13.6% were active smokers), and unmarried (Table 1). Since our study evaluates stress levels among medical students, we asked our participants if they live alone or with family/friends. Approximately 99.1% of the female participants and 94.6% of the males live with parents, siblings, or friends; while the rest live by themselves. None of our participants reported health issues that required hospital admission during this study.

**Table 1:** Sociodemographic, dietary and lifestyle behaviors, sleep, mental stress, and academic performance characteristics for the study participants (*n* = 220)

Variable	Value	
**Sex, n (%)**		
Female	109 (49.5)	
Male	111 (50.5)	
**Age, mean (SD)**	19.95 (1.73)	
**Academic year, n (%)**		
First	61 (27.73)	
Second	57 (25.91)	
Third	55 (25)	
Fourth	15 (6.82)	
Fifth	26 (11.82)	
Sixth	6 (2.73)	
**Marital status, n (%)**		
Married	5 (2.3)	
Unmarried	215 (97.7)	
**Smoking, n (%)**		
No	190 (86.4)	
Yes	30 (13.6)	
**Chronic illnesses**^**a**^**, n (%)**		
No	200 (90.9)	
Yes	20 (9.1)	
**BMI, mean (SD)**	23.31 (5.96)	
**MMDS, mean (SD)**	3.81 (1.33)	
**Physical activity, n (%)**		
≥ 3 times/week	47 (21.36)	
< 3 times/week	173 (78.64)	
**Sleep duration, n (%)**		
At least 7–8 h	90 (40.91)	
< 7 h	130 (59.09)	
**PSS, mean (SD)**	20.18 (5.44)	
**Academic performance, n (%)**		
A + /A	84 (38.13)	
B + /B	90 (40.91)	
C + /C	34 (15.45)	
D + /D	10 (4.55)	
F	2 (0.91)	

^a^chronic illnesses include any disease lasting for 3 months or longer which is under control without exacerbations throughout the study, and doesn’t interfere with sleep, mental health, and/or daily activity. BMI: body mass index. MMDS: modified Mediterranean diet score. Physical activity intensity: moderate-vigorous. PSS: perceived stress score

### Healthy dietary and lifestyle scores upon RDIF

Overall, RDIF is associated with better dietary and healthy lifestyle scores in both female and male students (Table 2). Compared with male students, the dietary and lifestyle score means seem to be higher among female students before and at the end RDIF, however, no statistically significant differences were observed. Linear regression was calculated to evaluate the relationship between dietary and lifestyle scores and academic year (reference group: first-year students). Our data showed that healthy dietary and lifestyle scores were associated with a decreased academic year before RDIF (Coefficient = -0.3; *P*-value < 0.01) and at the end of RDIF (Coefficient = -0.47; *P*-value < 0.05). The relationship between RDIF and dietary and lifestyle scores according to academic years was investigated. We found significant changes in dietary and lifestyle scores during Ramadan in first-year medical students, who showed the highest dietary and lifestyle scores before and at the end of RDIF, while the fourth-year students showed no changes in the average score and the sixth-year students had lower scores at the end of RDIF compared with pre-RDIF scores (Table 2). Before RDIF, most of our participants had shown a BMI < 25, were less adherent to the Mediterranean diet, exercised less than three times a week, and slept less than 7 h per day. However, they became more active and adherent to the Mediterranean diet while observing RDIF (Table 3). In addition, some of the smokers quit smoking during Ramadan (13.64% vs 6.63%, *P*-value < 0.01). Although not statistically significant, shorter sleep duration was observed during RDIF (sleep < 7 h before and after RDIF: 59.09% vs 63.64%, respectively). While female students were more susceptible to being overweight during RDIF, they became more active and adherent to the Mediterranean diet (Table 4). Students living alone had significantly lower dietary and lifestyle score mean during RDIF compared with other students living with family or friends (1.86 ± 0.7 vs 2.77 ± 1.1, *P*-value < 0.05).

**Table 2:** Dietary and lifestyle score pre- and post-RDIF according to sex and academic year^a^

	Pre-RDIF Mean (SD)	Post-RDIF Mean (SD)	Change score	*P*-value
**Overall**	2.42 (0.98)	2.74 (1.09)	0.32	< 0.05
**Sex**				
Female	2.51 (0.92)	2.80 (1.14)	0.29	< 0.05
Male	2.33 (1.04)	2.68 (1.05)	0.35	< 0.05
**Academic year**				
First	2.59 (1.32)	3.00 (1.11)	0.41	< 0.05
Second	2.40 (1.53)	2.72 (1.16)	0.32	NS
Third	2.44 (1.24)	2.78 (1.07)	0.34	NS
Fourth	2.27 (1.50)	2.27 (1.03)	0	NS
Fifth	2.27 (1.35)	2.62 (0.85)	0.35	NS
Sixth	1.83 (1.03)	1.67 (0.82)	-0.16	NS

^a^Total score is 5. Change score was calculated by subtracting the post-RDIF mean score from the pre-RDIF mean score. *P*-value was computed using Student’s t-test

**Table 3:** Dietary and lifestyle score components pre- and post-RDIF in the overall sample

	Pre-RDIF Frequency (%)	Post-RDIF Frequency (%)	P-value*
**Body Mass Index(BMI, kg/m**^**2**^**)**			
< 25	164 (74.55)	143 (65.00)	< 0.05
≥ 25	56 (25.45)	77 (35.00)	
**Adherence to the Mediterranean diet**			
≥ 5 points	42 (19.09)	71 (32.27)	< 0.01
< 5 points	178 (80.91)	149 (67.73)	
**Physical activity**			
≥ 3 times/week	47 (21.36)	92 (41.82)	< 0.001
< 3 times/week	173 (78.64)	128 (58.18)	
**Smoking**			
No	190 (86.36)	206 (93.64)	< 0.01
Yes	30 (13.64)	14 (6.36)	
**Sleep duration**			
At least 7–8 h	90 (40.91)	80 (36.36)	NS
< 7 h	130 (59.09)	140 (63.64)	

^*^P-value was computed using Chi-square test

**Table 4:** Dietary and lifestyle score components pre- and post-RDIF according to sex

	Before RDIF Frequency (%)		After RDIF Frequency (%)		*P*-value*	
	**Female**	**Male**	**Female**	**Male**	**Female**	**Male**
**Body Mass Index (BMI, kg/m**^**2**^**)**						
< 25	96 (88.07)	68 (61.26)	84 (77.06)	59 (53.15)	< 0.05	NS
≥ 25	13 (11.93)	43 (38.74)	25 (22.94)	52 (46.85)		
**Adherence to Mediterranean Diet**						
≥ 5 points	18 (16.51)	24 (21.62)	32 (29.36)	39 (35.14)	< 0.05	NS
< 5 points	91 (83.49)	87 (78.38)	77 (70.64)	72 (64.86)		
**Physical activity**						
≥ 3 times/week	17 (15.60)	30 (27.03)	43 (39.45)	49 (44.14)	< 0.001	< 0.01
< 3 times/week	92 (84.40)	81 (72.97)	66 (60.55)	62 (55.86)		
**Smoking**						
No	100 (91.74)	90 (81.08)	103 (94.50)	103 (92.79)	NS	< 0.05
Yes	9 (8.26)	21 (18.92)	6 (5.50)	8 (7.21)		
**Sleep duration**						
At least 7–8 h	52 (47.71)	38 (34.23)	47 (43.12)	33 (29.73)	NS	NS
< 7 h	57 (52.29)	73 (65.77)	62 (56.88)	78 (70.27)		

^*^P-value was computed for comparisons between the two time points for each sex

### Perceived stress scale (PSS) scores upon RDIF

The overall average of PSS scores before observing RDIF was 20.18. The PSS scores were higher during RDIF, especially in male students who exhibited lower PSS scores than females before RDIF (Table 5). Significant changes in PSS scores were seen in the 4^th^ and 5^th^-year students. However, the first- and sixth-year students showed the least PSS score changes. The 5^th^-year students had the lowest PSS average score before RDIF while the 4^th^-year students had the highest PSS scores during RDIF. To check if PSS is associated with dietary and lifestyle components, linear regression analysis was performed. PSS score was denoted as the outcome (dependent) variable while dietary and lifestyle components were the exposures (independent variables). No association was found between PSS and healthy dietary and lifestyle elements (Supplementary Table 3).

**Table 5:** Perceived Stress Scale (PSS) pre- and post- RDIF according to sex and academic year^a^

	Pre-RDIF Mean (SD)	Post-RDIF Mean (SD)	Change score	*P*-value
**Overall**	20.18 (5.44)	21.84 (5.48)	1.66	< 0.01
**Sex**				
Female	20.85 (5.36)	21.62 (4.96)	0.77	NS
Male	19.52 (5.47)	22.05 (5.95)	2.53	< 0.01
**Academic year**				
First	21.41 (4.80)	21.97 (4.95)	0.56	NS
Second	20.33 (5.67)	21.98 (5.18)	1.65	NS
Third	19.51 (5.93)	21.16 (6.17)	1.65	NS
Fourth	21.40 (3.64)	24.80 (4.83)	3.40	< 0.05
Fifth	17.73 (5.90)	21.31 (6.01)	3.58	< 0.05
Sixth	20.00 (3.41)	20.33 (5.01)	0.33	NS

^a^Total score is 40. Change score was calculated by subtracting the post-RDIF mean score from the pre-RDIF mean score. *P*-value was computed using Student's t-test

### Body mass index (BMI) and academic performance during RDIF

To investigate the impact of RDIF on BMI, BMI means and standard deviations were calculated before and at the end of observing RDIF. No significant differences were observed in the overall sample or according to sex or academic year (Supplementary Table 4). However, the number of overweight/obese female students has increased during Ramadan month (Table 4). There were no significant differences in the last exam grades between the two-time points, i.e., before and during RDIF (Supplementary Table 5). Moreover, no changes in academic performance were seen according to sex or academic year (not shown here).

## Discussion

Recently, IF has become a popular eating plan to lose weight and might be an effective treatment for overweight and obesity [40]. Furthermore, current evidence indicates that RDIF has beneficial effects on cardiometabolic health [1, 2]. However, little is known about its effects on the lifestyle, stress levels, and productivity of healthy young adults. In this longitudinal study, we assessed healthy lifestyle factors, perceived stress levels, and academic performance in medical students.

Our data suggest a healthier lifestyle among medical students during Ramadan. However, no effects of RDIF on BMI means have been reported. This is consistent with a recent systematic review that found no significant differences in the BMI of people with normal weight [2]. However, another study found a slight decrease in body weight of non-athlete healthy individuals [41]. In our study, the number of our students who became overweight (BMI ≥ 25) has increased at the end of Ramadan, especially females. This could be explained by the possible variations in eating patterns and food compositions based on geographical locations and culture [42]. Nevertheless, during RDIF, our participants were more adherent to a Mediterranean diet, which is linked to a lower risk of cardiovascular disease, increased lifespan, and better sleep quality in university students [43, 44].

Our participants were more physically active during Ramadan. In contrast, a recent study found an association between reduced physical activity and RDIF in adults. However, there were no significant differences when data were stratified by age groups [45]. Moreover, that study was conducted on the general population aged 25 years or above; while our study included only medical students with an age range of 18 to 25 years. Reduction in smoking behavior during Ramadan has been reported in previous studies [46, 47]. This is consistent with our data that showed more than one-third of male smokers had quit smoking. While the aforementioned dietary and lifestyle components showed some differences upon RDIF, sleep duration remained unchanged throughout Ramadan. Similarly, no effects of RDIF on sleep duration have been reported in an objectively assessed sleep patterns study [9].

RDIF showed beneficial effects on psychological well-being and depressive symptoms [48]. This could be explained by the ameliorating effect of RDIF on the pro-inflammatory cytokines, such as interleukin-6 (IL-6) and tumor necrosis factor- α (TNF- α) which contribute to the pathophysiology of depression [49, 50]. Perceived stress was higher among our male participants during Ramadan. Before RDIF, male students had lower PSS than females. This is consistent with a previous study conducted among college students [51]. At the end of RDIF, we observed significant changes in PSS only in males. This could be explained by the significant reductions in testosterone levels upon RDIF [52]. It has been reported previously that lower testosterone levels is associated with depressive symptoms and anxiety [53]. There was no correlation between PSS and dietary and lifestyle components, suggesting that stress levels in these students might be affected mainly by RDIF. It has been reported that short-term fasting is accompanied by increased negative emotions, such as irritability and depression. This suggests that IF could be associated with negative mood and stress. While this might be uncommon with religious fasting such as RDIF, several factors might have contributed to the increased perceived stress levels among the students, such as observing the first Ramadan at school after the COVID-19 lockdown. In fact, experts in psychology and psychiatry claimed that the mental health impact of COVID-19 might last for several years [54, 55].

RDIF didn’t affect the academic performance of our participants. Nonetheless, RDIF is associated with impaired academic achievement [20]. An explanation for this contradictory result is that our study was conducted in Saudi Arabia where school days are shortened during Ramadan. Another reason is using a categorical marking system which might obscure differences in numerical grades, such as GPA. Consequently, further studies are needed to evaluate the impact of IF on the academic productivity of school and university students.

To the best of our knowledge, this is the first study investigating the effects of RDIF on the general well-being of medical students. A key advantage of this longitudinal study is the ability to identify changes over time in the same population. These findings may help to develop guidelines and recommendations for those observing RDIF or following IF plans for health benefits. However, our study is limited by sample size, especially in the fourth- and sixth-year students, and doesn’t prove causality. This study was conducted in Taif university among medical students and cannot be generalized to all other students, countries and/or other cultures. Furthermore, our data were obtained via self-reported questionnaires and subjective assessment tools. Therefore, future research would involve objective assessments of the parameter studied and larger sample recruitment.

Overall, RDIF is associated with a healthier diet and lifestyle in medical students and may not affect their academic performance, with noticeable perceived stress being higher in male students.

## Conclusion

This study assessed changes in dietary and lifestyle, stress levels, and academic performance among medical students, who are vulnerable to high-stress levels, in a Muslim country. We found an improvement in lifestyle, such as consuming a healthy diet, increasing physical activity, and smoking cessation. This suggests that RDIF can promote healthy behaviors during Ramadan in a Muslim country. However, perceived stress was higher among male students. While this is not expected, observing the first Ramadan after the COVID-19 lockdown might be one of the reasons. Finally, our data showed no impact of RDIF on students’ performance in exams.

## Supplementary Information

**Additional file 1: Supplementary Table 1. **Modified Mediterranean Diet Score (MMDS). **Supplementary Table 2.** Perceived Stress Scale (PSS).  **Supplementary Table 3.** Linear regression analysis of the relationship between perceived stress scale (PSS) scores (outcome variable) and lifestyle components. **Supplementary Table 4.** BMI average pre- and post- RDIF according to sex and academic year. **Supplementary Table 5.** Academic performance pre- and post- RDIF in overall sample. 

## Data Availability

Data is available upon request. Please contact the corresponding author/s for further inquiries.

## Abbreviations

RDIF: Ramadan diurnal intermittent fasting
MMDS: Modified Mediterranean diet scores
PSS: Perceived stress scale
BMI: Body mass index
COVID-19: Coronavirus Disease 2019
