# Supplementary material for: Changes in dietary and lifestyle behaviors and mental stress among medical students upon Ramadan diurnal intermittent fasting: a prospective cohort study from Taif/Saudi Arabia
Source: BMC Public Health. 2023 Jul 31;23:1462. doi: 10.1186/s12889-023-16385-1 (PMC10391762; doi:10.1186/s12889-023-16385-1)
Supplement: Supplementary file 1 — Additional file 1: Supplementary Table 1. Modified Mediterranean Diet Score (MMDS). Supplementary Table 2. Perceived Stress Scale (PSS). Supplementary Table 3. Linear regression analysis of the relationship between perceived stress scale (PSS) scores (outcome variable) and lifestyle components. Supplementary Table 4. BMI average pre- and post- RDIF according to sex and academic year. Supplementary Table 5. Academic performance pre- and post- RDIF in overall sample. [file 12889_2023_16385_MOESM1_ESM.docx]

***Supplementary Table 1.*** Modified Mediterranean Diet Score (MMDS).

| Questions | Answers | Score |
| --- | --- | --- |
| How much olive oil do you consume per day? | < 4 teaspoons | 0 |
|  | ≥ 4 teaspoons | 1 |
| How many vegetable servings do you consume per day? (Serving size= 200g) | < 2 serving size | 0 |
|  | ≥ 2 serving size | 1 |
| How many fruit units do you consume per day? | < 3 units or serving size of natural juice | 0 |
|  | ≥ 3 units or serving size of natural juice | 1 |
| How many servings of red meat or meat products do you consume per day? | ≥ one serving size | 0 |
|  | < one serving size | 1 |
| How many servings of butter, margarine, or cream do you consume per day? | ≥ one serving size | 0 |
|  | < one serving size | 1 |
| How many sweets or carbonated beverages do you consume per day? | ≥ one serving size | 0 |
|  | < one serving size | 1 |
| How many servings of fish or shellfish do you consume per week? | < 3 serving size | 0 |
|  | ≥ 3 serving size | 1 |
| How many times per week do you consume commercial sweets or pastries (not homemade), such as cakes, cookies, biscuits, or  custard? | ≥ 3 times | 0 |
|  | < 3 times | 1 |
| How many servings of legumes do consume per week? (1 serving= 150g) | < 3 times | 0 |
|  | ≥ 3 serving size | 1 |
| How many servings of nuts (including peanuts) do you consume per week? | < 3 serving size | 0 |
|  | ≥ 3 serving size | 1 |

*****Serving size is often printed on nutrition labels and refers to the FDA 's recommended amount of a particular food you would normally eat in one sitting.

***Supplementary Table 2.*** Perceived Stress Scale (PSS)

| Questions | Never | Almost never | Sometimes | Fairly often | Very often |
| --- | --- | --- | --- | --- | --- |
| 1. In the last month, how often have you been upset because of something that happened unexpectedly? | 0 | 1 | 2 | 3 | 4 |
| 1. In the last month, how often have you felt that you were unable to control the important things in your life? | 0 | 1 | 2 | 3 | 4 |
| 1. In the last month, how often have you felt nervous and “stressed”? | 0 | 1 | 2 | 3 | 4 |
| 1. In the last month, how often have you felt confident about your ability to handle your personal problems? | 0 | 1 | 2 | 3 | 4 |
| 1. In the last month, how often have you felt that things were going your way? | 0 | 1 | 2 | 3 | 4 |
| 1. In the last month, how often have you found that you could not cope with all the things that you had to do? | 0 | 1 | 2 | 3 | 4 |
| 1. In the last month, how often have you been able to control irritations in your life? | 0 | 1 | 2 | 3 | 4 |
| 1. In the last month, how often have you felt that you were on top of things? | 0 | 1 | 2 | 3 | 4 |
| 1. In the last month, how often have you been angered because of things that were outside of your control? | 0 | 1 | 2 | 3 | 4 |
| 1. In the last month, how often have you felt difficulties were piling up so high that you could not overcome them? | 0 | 1 | 2 | 3 | 4 |

| **Supplementary Table 3. Linear regression analysis of the relationship between perceived stress scale (PSS) scores (outcome variable) and lifestyle components** | | | | | | | | | | | | | | | | | | | | |
| --- | --- | --- | --- | --- | --- | --- | --- | --- | --- | --- | --- | --- | --- | --- | --- | --- | --- | --- | --- | --- |
| **Before RDIF** | | | | | | | | | | **After RDIF** | | | | | | | | |  |  |
| **Variable**  **(Reference group)** | | | | | **Coefficient** | | | **P-value** | | | | | | **Coefficient** | | | **P-value** | |  |  |
| **BMI (≥ 25)** | | | | | | | | | | | | | | | | | | | |  |
| < 25 | | 0.002 | | | NS | | | | | | 0.01 | | | | NS | | |  |  |  |
| **Adherence to MMDS**  **(< 5 points)** | | | | | | | | | | | | | | | | | | | |  |
| ≥ 5 points | 0.0001 | | | NS | | | | | | | 0.002 | | | NS | | |  |  |  |  |
| **Physical activity**  **(< 3 times/week)** | | |  | | | | | |  | | | | | | | | | | |  |
| ≥ 3 times/week | | 0.009 | | NS | | | | | | | 0.02 | | NS | | | |  |  |  |  |
| **Smoking**  **(Yes)** | | |  | | |  | | | | |  | | | | | | | |  |  |
| No | 0.004 | | | | NS | | | | | | 0.001 | | | NS | |  |  |  |  |  |
| **Sleeping duration**  **(< 7 h)** | | |  | | |  | | | | |  | | | | | | | |  |  |
| At least  7-8 h | | 0.002 | | | | NS | | | | | 0.01 | | | NS | |  |  |  |  |  |

| **Supplementary Table 4. BMI average pre- and post- RDIF according to sex and academic year** | | | | | | | | | | | | |
| --- | --- | --- | --- | --- | --- | --- | --- | --- | --- | --- | --- | --- |
| **Pre-RDIF**  **Mean (SD)** | | | | | | | **Post-RDIF**  **Mean (SD)** | | | **P-value** | | |
| **Overall** | | | 22.78 (5.4) | | | | 23.31 (5.96) | | | NS | | |
| **Sex** | | | | | | | | | | | | |
| Female | | 20.77 (3.49) | | | | 21.5 (4.55) | | NS | | | |  |
| Male | | 24.76 (6.22) | | | | 25.09 (6.63) | | | | NS |  |  |
| **Academic year** | | | | | | | | | | | | |
| First | 21.95 (5.35) | | |  | | 23.16 (6.56) | | | NS | |  |  |
| Second | 22.74 (5.79) | | |  | | 22.59 (5.65) | | | NS | |  |  |
| Third | 22.03 (4.09) | | |  | | 22.44 (5.47) | | | NS | |  |  |
| Fourth | 24.45 (6.52) | |  | | 26.37 (6.66) | | | NS | | |  |  |
| Fifth | 24.58 (6.10) |  | | | | 24.72 (5.44) | | | NS | |  |  |
| Sixth | 26.65 (5.05) |  | | | | 26.39 (4.60) | | | NS | |  |  |

| **Supplementary Table 5. Academic performance pre- and post- RDIF in overall sample** | | | | | | | | | |
| --- | --- | --- | --- | --- | --- | --- | --- | --- | --- |
| **Pre-RDIF**  **Frequency (%)** | | | | | | **Post-RDIF**  **Frequency (%)** | | **P-value** | |
| **Last exam grade** | | | | | | | | | |
| A+/A | 84 (38.18) | | |  | | 97 (44.09) | | NS |  |
| B+/B | 90 (40.91) | | |  | | 78 (35.45) | | NS |  |
| C+/C | 34 (15.45) | | |  | | 34 (15.45) | | NS |  |
| D+/D | 10 (4.55) | |  | | 10 (4.55) | | NS | |  |
| F | 2 (0.91) |  | | | | 1 (0.45) | | NS |  |
